# Current status and emerging trends in biological therapies for ankylosing spondylitis: A bibliometric analysis (2004–2024)

**DOI:** 10.1097/MD.0000000000044201

**Published:** 2026-01-23

**Authors:** Rong Deng, Xuan Jiang, Xin-xin Zhong, Fu Shen, Feng Qian, Chang-wen Quan

**Affiliations:** a Department of Orthopedics and Traumatology, Yongzhou Hospital of Traditional Chinese Medicine, Yongzhou, China; b Department of Dermatology, Yongzhou Hospital of Traditional Chinese Medicine, Yongzhou, China.

**Keywords:** ankylosing spondylitis, bibliometrics, biological therapies, CiteSpace

## Abstract

**Background:**

Ankylosing spondylitis (AS) is a chronic inflammatory disease characterized by pain and stiffness in the spine and sacroiliac joints. It significantly reduces quality of life and imposes substantial social and economic burdens. Advances in understanding AS have led to the development of biological therapies, including tumor necrosis factor-α inhibitors, interleukin-17 inhibitors, and Janus kinase (JAK) inhibitors, which show promise in managing symptoms and improving patient outcomes. This study aims to explore the overall research trends and future directions in this field using bibliometric methods.

**Methods:**

This study conducted a bibliometric analysis of research on biological therapies for AS from 2004 to 2024. We searched the Web of Science Core Collection and PubMed databases for relevant studies and used tools such as Microsoft Excel, CiteSpace, and VOSviewer to analyze publication trends, citation patterns, and research hotspots. The analysis focused on identifying key research areas, influential publications, and emerging trends in the field.

**Results:**

Our findings revealed a significant increase in publications, citations, and scholarly involvement over the past 2 decades. The United States, China, and Germany were the leading countries in publication numbers. Key research areas included the efficacy of biological agents, personalized treatment strategies, and combination therapies. The most cited studies focused on the clinical impact of tumor necrosis factor-α inhibitors, interleukin-17 inhibitors, and emerging therapies such as Janus kinase inhibitors. Future research is expected to explore dual-target biologics, new cytokine pathways, gene therapy, and the impact of biological therapies on the gut microbiota.

**Conclusion:**

This bibliometric analysis highlights the growing interest in biological therapies for AS, identifying research gaps and future directions to enhance treatment and improve patient outcomes. The findings underscore the need for further research on the long-term efficacy and safety of biological agents and the development of personalized and combination therapies to address the complex challenges posed by AS.

## 1. Introduction

Ankylosing spondylitis (AS) is a chronic inflammatory disease affecting the spine and sacroiliac joints, causing pain and stiffness, primarily in young males.^[1]^ Its exact cause is unknown, but genetics, especially the human leukocyte antigen B27 (HLA-B27) gene, play a key role.^[2]^ AS is more prevalent among white populations in Northern Europe and North America and less common in Asian and African groups.^[3]^ In China, the prevalence is 0.2% to 0.5%, with more males affected than females.^[4]^ AS significantly impacts quality of life by limiting mobility and causing pain, fatigue, stress, and depression.^[5]^ Furthermore, individuals with AS frequently experience fatigue, stress, and depression, which diminishes their quality of life.^[6]^ AS can reduce a patient’s work capacity and increase financial strain.^[7]^ Research indicates that compared with the general population, AS patients have a lower quality of life, particularly in terms of physical and mental health.^[8]^ Comprehensive management, including medication, physical therapy, and psychological support, enhances their quality of life.^[9]^ Early diagnosis and effective treatment can lessen the adverse effects of AS and improve patients’ overall health.

Conventional treatments for AS, such as nonsteroidal anti-inflammatory drugs (NSAIDs) and conventional synthetic disease-modifying antirheumatic drugs, have limitations in controlling disease activity and structural damage.^[10]^ NSAIDs are ineffective in preventing bone mineral formation. In contrast, conventional synthetic disease-modifying antirheumatic drugs such as sulfasalazine and methotrexate primarily address peripheral arthritis but have little effect on spinal progression.^[11]^ Advances in understanding AS have led to the development of biological therapies, with tumor necrosis factor-α (TNF-α) inhibitors showing promise in managing inflammatory pain and some extra-articular symptoms. However, their impact on slowing spinal progression is debated, with mixed study results.^[12]^ Biologics that target interleukin-17 (IL-17) and interleukin-23 (IL-23), such as secukinumab, offer promising alternatives for AS patients who do not respond to TNF-α inhibitors.^[13]^ These therapies have improved symptom control and opened new avenues for long-term disease management.^[14]^ However, further research is needed to assess their long-term efficacy and safety across different populations.^[15]^ The high cost of biologics remains a barrier, highlighting the need for more affordable treatment options.

Bibliometrics is a discipline that analyzes the quantitative characteristics of literature – such as academic papers, books, and journals – and their interrelationships. By employing quantitative analyses of indicators such as publication output, research themes, and citations, bibliometrics reveals the developmental dynamics, research trends, and knowledge dissemination characteristics within academic disciplines. Despite extensive research on biological agents for treating AS, the general trends in research within this field remain poorly defined. The present study seeks to thoroughly investigate the current research developments and areas of growing interest in the use of biologic agents for AS from a bibliometric perspective, thereby providing valuable insights for upcoming studies in this domain.

## 2. Methods

### 2.1. Sources of data and search methodologies

**Figure 1.**
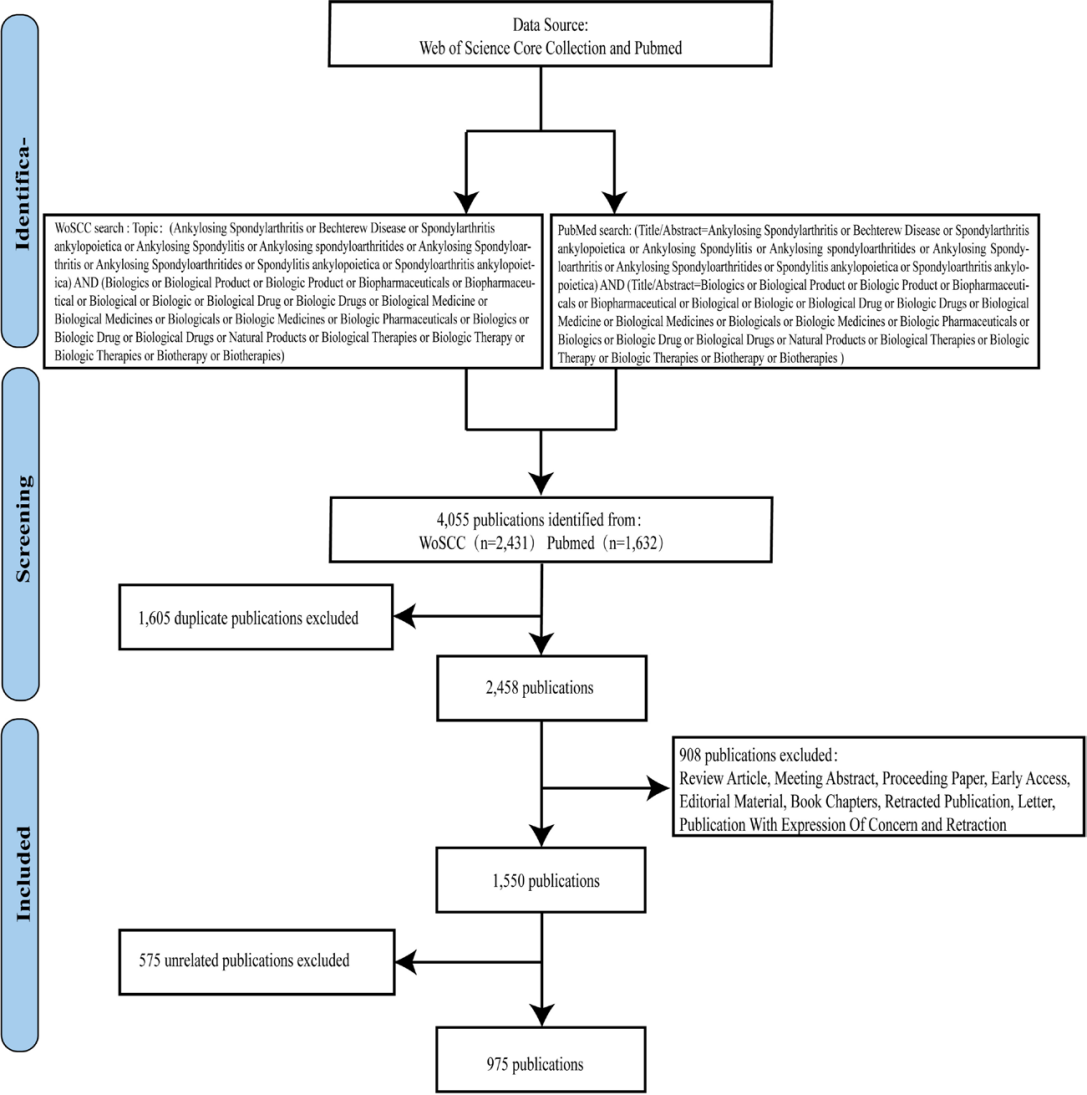
Flowchart illustrating the literature search and screening procedure. 4055 publications were identified through the search strategy from the WoSCC (2431 publications) and PubMed (1632 publications). After removing duplicates (1605 publications) and excluding review articles, meeting abstracts, proceedings papers, early access articles, editorial materials, book chapters, retractions, letters, publications with expressions of concern, and retractions (908 publications), as well as irrelevant publications (575 publications), a final total of 975 publications were included in the study. WoSCC = Web of Science Core Collection.

We searched PubMed and the Web of Science Core Collection for all studies on biological therapies and AS published between January 1, 2004, and August 31, 2024. On October 1, 2024, we completed all searches to prevent bias in the quantity of documents resulting from database upgrades. We used the terms “biological therapies” and “ankylosing spondylitis” (as well as their Medical Subject Headings synonyms). Rong Deng and Xuan Jiang independently screened and excluded studies in this study. Xin-xin Zhong reviewed any discrepancies and made the final decisions. Figure 1 illustrates the flowchart of the literature search and screening process.

### 2.2. Data collection and analysis

The annual publication volume was imported into Microsoft Office Excel 2021 to analyze the yearly output of the articles. VOSviewer 1.6.20 was utilized to construct co-occurrence and timeline maps for countries/regions, journals, and authors. Additionally, CiteSpace 6.3 R3 was employed to create journal dual overlay maps, co-citation reference maps, reference burst maps, keyword clusters, keyword timeline maps, and keyword burst maps. Furthermore, we extracted bibliometric data, including publication volume for countries, journals, authors, cited documents, and frequently occurring keywords.^[16]^

## 3. Results

### 3.1. Annual number of publications/citations/scholars

**Figure 2.**
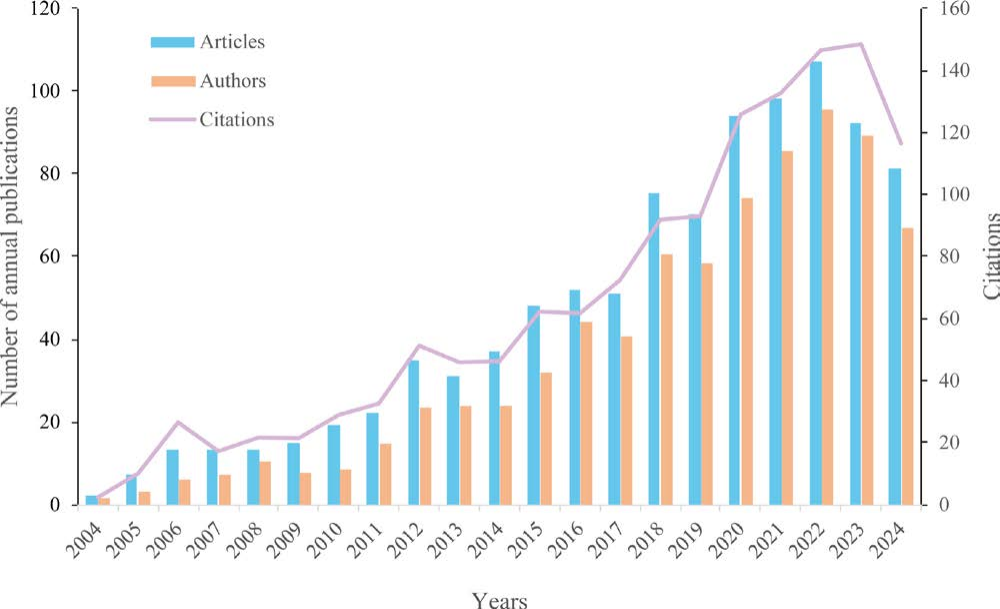
Trends in the global annual number of publications, number of authors (actual values multiplied by 0.1), and total citations (actual values multiplied by 0.1). “Articles” refers to the total number of publications about ankylosing spondylitis biotherapy. “Authors” represents the number of authors contributing to these publications. “Citations” indicates the total number of citations of these publications.

A total of 975 publications were included in the biotherapy for AS. Through a statistical analysis of “authors,” “organizations,” “countries,” and “sources” using VOSviewer 1.6.20, it was found that these works were authored by 5596 authors from 2178 institutions across 81 countries and regions and were published in 249 different journals. Furthermore, the publications referenced 19,906 articles from 3997 distinct journals. Figure 2 shows the global trends in yearly publications, the number of scholars, and the total number of citations in AS biotherapy. The figure illustrates a significant increase in research activity within the field of biological therapies for AS over the past 2 decades. There has been a rising trend in research studies, citation counts, and the number of contributing scholars. Although the data for 2024 include only relevant literature published from January 1 to August 31, the publication volume is approaching that of 2023. This pattern indicates that the importance of biologically targeted therapies in AS research has garnered widespread attention, establishing them as a significant field of study in recent years.

### 3.2. Countries/regions analysis

**Figure 3.**
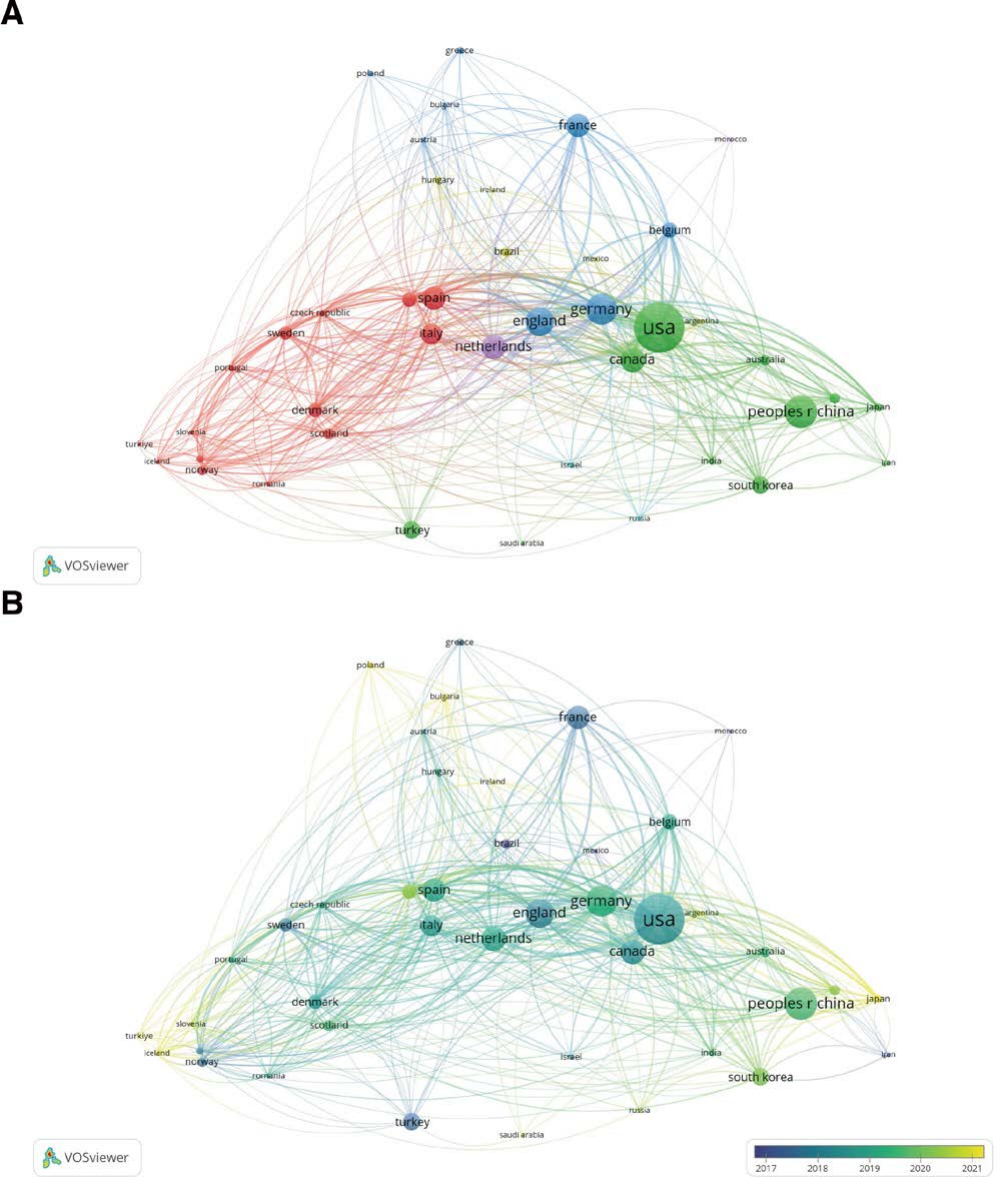
(A) Cooperation network of countries/regions related to publications on AS biotherapy. The size of the nodes represents the volume of publications, with larger nodes indicating a higher number of publications. Nodes of the same color signify a highly collaborative cluster. The curves represent the collaboration between countries/regions, with thicker curves indicating closer cooperation. (B) Overlay visualization map of countries involved in the study of AS biotherapy. The color represents the average year a country published literature, with blue representing countries that have researched biological therapies for AS earlier than those described by yellow. AS = ankylosing spondylitis.

A total of 81 countries contributed 975 papers on biotherapy for AS. Table 1 presents the top 10 countries by publication count. The United States recorded the highest output, with 214 publications (21.95%), followed by China with 123 publications (12.62%), Germany with 116 publications (11.90%), and England with 104 publications (10.67%). Figure 3A illustrates the cooperation network among countries and regions engaged in research on biotherapy for AS. This map includes 43 countries and regions, each having published at least 5 articles. The United States has the highest level of collaboration, with 37 connections, followed closely by England (36 connections), Germany (35 connections), Spain (35 connections), and Italy (35 connections). The overlay visualization map of countries illustrates the average year each country published articles in AS biotherapy (Fig. 3B). Different colors are assigned to the countries based on the average publication year, thereby highlighting the primary contribution periods of various nations. Over time, countries in blue published earlier than those in yellow, indicating a temporal distinction in their contributions to developing biotherapy for AS. As shown in Figure 3B, countries including Mexico, Brazil, Iran, Turkey, and France made significant contributions to this field before 2019. Countries such as Japan, Bulgaria, and Argentina have recently contributed substantially to this subject. In summary, among the 81 contributing countries, the United States leads in publication output and collaboration. At the same time, notable contributions have emerged from countries such as Japan, Bulgaria, and Argentina in recent years, highlighting the evolving research landscape in this field.

**Table 1:** Top 10 countries/regions research on biotherapy for ankylosing spondylitis.

Country	Counts	%	Citations	Avg. citations	Links
USA	214	21.95	7922	37.02	37
PR China	123	12.62	2051	16.67	22
Germany	116	11.90	5438	46.88	35
England	104	10.67	3413	32.82	36
Canada	88	9.03	3840	43.64	33
Netherlands	88	9.03	4703	53.44	33
Spain	82	8.41	2407	29.35	35
France	81	8.31	2140	26.42	25
Italy	71	7.28	1885	26.55	35
South Korea	59	6.05	1791	30.36	24

“Counts” indicates the number of publications from that country/region. “%” indicates the percentage of total publications that country/region contributed. “Citations” refers to the total number of citations received by publications from that country/region. “Avg. citations” represents the average number of citations per publication from that country/region. “Links” refers to the number of countries/regions collaborating with the listed country/region in research publications.

### 3.3. Journal analysis

**Figure 4.**
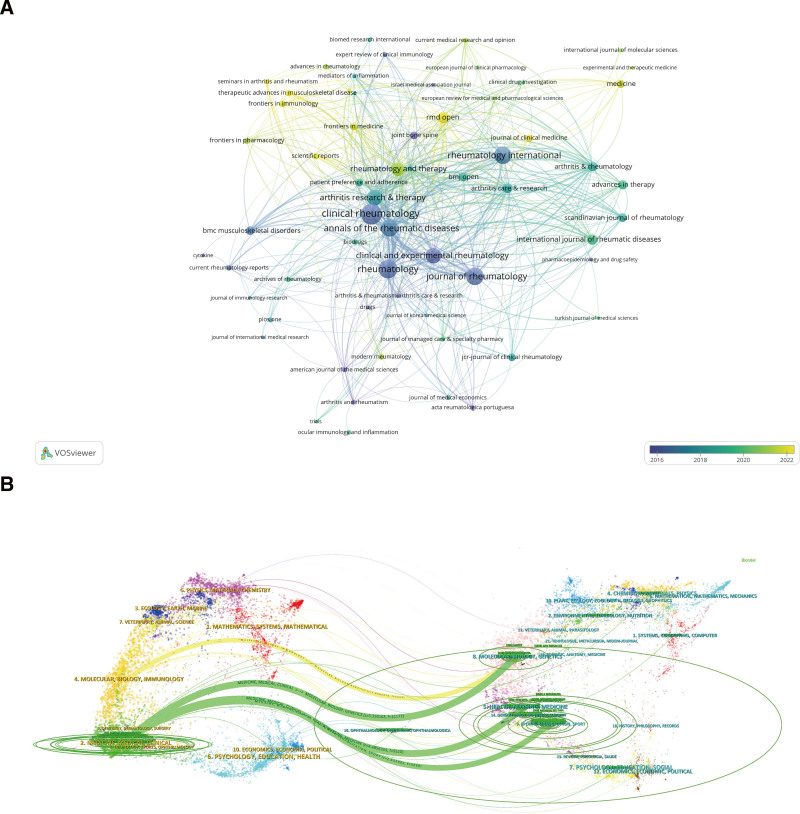
(A) Overlay visualization of journal maps. The color represents the average year in which a journal publishes literature. Journals represented by blue publish papers on biotherapy for AS earlier than those represented by yellow. (B) Dual-map overlay of journals. This dual-map illustrates the citing and cited relationships among journals in AS biotherapy. The left side shows citing journals, predominantly from medicine and clinical fields, while the right displays frequently cited journals. The different colored labels represent the disciplines covered by the journal. AS = ankylosing spondylitis.

VOSviewer is used to analyze journal output, citations, and co-citations of journals (Fig. 4A and Table 2), whereas CiteSpace is employed to generate a dual-map overlay of periodicals (Fig. 4B). Table 2 presents a selection of the 10 foremost journals in AS biotherapy regarding output and co-citation frequency. The journal that published the most articles in this field was Clinical Rheumatology (61 articles), followed by Rheumatology (53 publications) and Rheumatology International (48 publications). Ann Rheum Dis boasts the highest impact factor among the 10 most output journals. The overlay visualization map of journals shows that, in recent years, there has been a growing trend of theses in this field being published in comprehensive journals, including but not limited to the Journal of Clinical Medicine, RMD Open, Frontiers in Immunology, and Medicine. Furthermore, the 3 journals with the highest co-citation frequencies are Annals of the Rheumatic Diseases, Journal of Rheumatology, and Arthritis & Rheumatology. The dual-map overlay of periodicals illustrates a concentration of citing and cited journals within this field (see Fig. 4B). The citing journals predominantly originate from the Medicine, Medical/Clinical, and Molecular Biology/Immunology categories. In contrast, the cited periodicals chiefly belong to the domains of Molecular Biology/Genetics, Health/Nursing/Medicine, and Sports/Rehabilitation/Sport. In summary, Clinical Rheumatology is the leading journal in article output, while recent trends indicate an increasing publication rate in comprehensive journals. The highest co-citation frequency is Annals of the Rheumatic Diseases.

**Table 2:** Top 10 journals on publication output or co-citations.

Rank	Journals	Documents	Citations	IF (2023)	Journals	Co-citations	IF (2023)
1	Clinical Rheumatology	61	1010	2.9	Annals of the Rheumatic Diseases	5276	20.29
2	Rheumatology	53	1468	4.7	Journal of Rheumatology	2002	3.59
3	Rheumatology International	48	710	3.19	Arthritis and Rheumatism	1675	8.95
4	Journal of Rheumatology	47	906	3.59	Arthritis & Rheumatology	1628	11.39
5	Annals of the Rheumatic Diseases	45	3367	20.29	Rheumatology	1583	4.7
6	Arthritis Research & Therapy	38	1796	4.39	Arthritis Research & Therapy	901	4.39
7	Clinical and Experimental Rheumatology	37	567	3.4	Lancet	793	98.4
8	Rheumatology and Therapy	29	268	2.89	Clinical and Experimental Rheumatology	597	3.4
9	RMD Open	23	187	5.1	Clinical Rheumatology	595	2.9
10	International Journal of Rheumatic Diseases	19	217	2.4	Arthritis Care & Research	592	3.7

“Documents” indicates the total number of publications in the journal. “Citations” indicates the total number of citations for journal publications. “IF (2023)” refers to the journal’s impact factor for the year 2023. “Co-citations” represents the number of times the journal has been co-cited with other journals.

### 3.4. Scholars analysis

**Figure 5.**
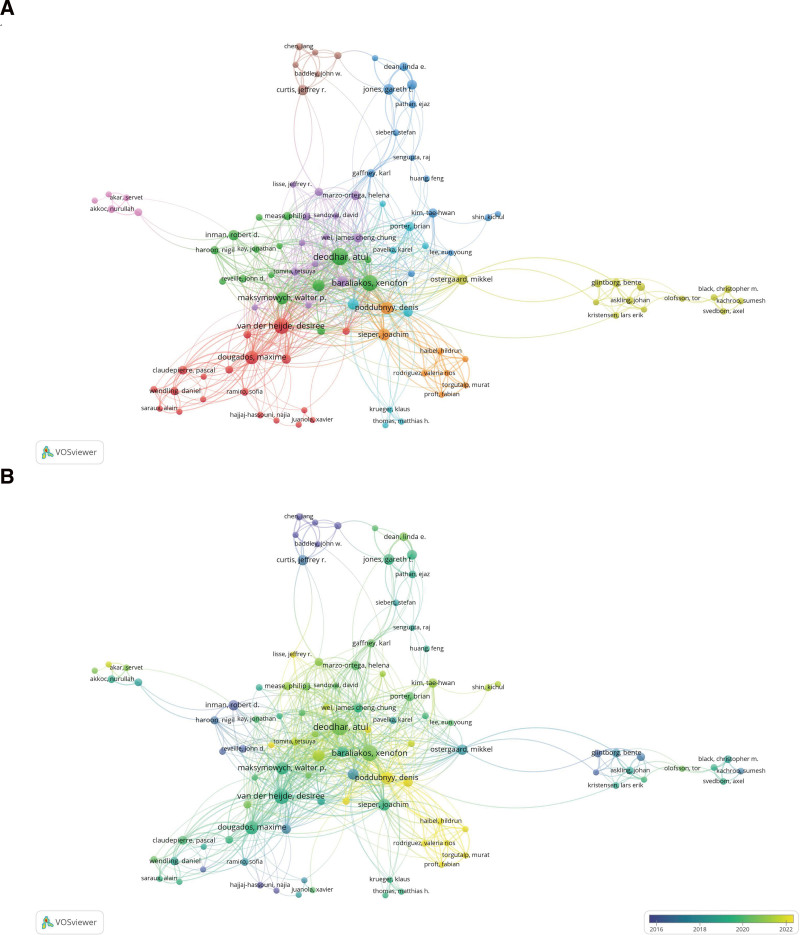
(A) Author collaboration network in the research of biologic therapies for AS (publications ≥ 5). Nodes represent authors, while the curves indicate collaborations between them. Nodes of the same color signify a closer collaboration cluster. (B) Author visualization overlay graph for the AS biotherapy study. The color of the nodes represents the publication timeline, with blue indicating authors who published articles on biological therapies for AS earlier than those represented in yellow. AS = ankylosing spondylitis.

A total of 5596 scholars have contributed to research on biotherapy for AS. The 3 authors with the most significant number of published papers in this sector are Deodhar A from Oregon Health & Science University (39 publications), Baraliakos X from Ruhr University Bochum (33 publications), and van der Heijde D from Leiden University Medical Center (32 publications), as shown in Table 3. Braun J from Ruhr University Bochum has the highest average number of citations per publication (Table 3). According to Price’s Law, scholars who have published at least 5 works (*T* ≥ 5) are regarded as core scholars in this field. A total of 102 such scholars were included in the creation of the network map of key authors (Fig. 5A). The overlay visualization map of the authors depicts the average publication year for each author in the field of AS biotherapy (Fig. 5B). Pioneering researchers in this area include Hajjaj-Hassouni N, Chen L, Inman RD, and Haroon N. More recently, significant contributions have been made by the authors Lisse JR, Poddubnyy D, Haibel H, and Navarro-Company V.

**Table 3:** Top 10 prolific authors in ankylosing spondylitis biotherapy research.

Rank	Author	Counts	Institutions	Citations	Avg. citations
1	Deodhar A	39	Oregon Health & Science University	1833	47.00
2	Baraliakos X	33	Ruhr University Bochum	1153	34.94
3	van der Heijde D	32	Leiden University Medical Center	2479	77.47
4	Dougados M	23	Rheumatology, Hospital Cochin	733	31.87
5	Maksymowych WP	21	University of Alberta	1594	75.90
6	Poddubnyy D	21	Charité – Universitätsmedizin Berlin	232	11.05
7	Gensler L	18	University of California San Francisco	937	52.06
8	Sieper J	18	Charité – Universitätsmedizin Berlin	1555	86.39
9	Jones GT	16	University of Aberdeen	331	20.69
10	Braun J	15	Ruhr University Bochum	1371	91.40

“Counts” refers to the total number of publications by the author. “Institutions” indicates the primary institutions associated with the author. “Citations” identifies the total number of citations received by the author’s publications. “Avg. citations” represents the author’s average number of citations per publication.

### 3.5. Research focal points analysis

#### 3.5.1. Most cited publications and citation burst references

The 10 most frequently cited publications focus on clinical research in this area (Table 4). The first is a report published by Machado et al in 2011 in the Annals of Rheumatic Diseases, which defines cutoff values for AS disease activity states and improvement scores.^[17]^ The second most cited publication is a randomized controlled trial by Baeten et al^[18]^ This study evaluated the effectiveness and safety of the anti-IL-17A monoclonal antibody secukinumab in treating patients with active AS. Ranking third is a recommendation update article published by Ward et al in 2019 in Arthritis & Rheumatology.^[19]^ This article presents similar recommendations for AS patients and nonradiographic axial spondyloarthritis (axSpA) patients. TNF inhibitors should be preferred over secukinumab or ixekizumab as the first choice for biologic treatment. Furthermore, discontinuing or tapering biological therapies is advised for patients with stable disease.

**Table 4:** Top 10 most globally cited documents in the field of ankylosing spondylitis biotherapy.

Rank	Documents	Citations	First author	Journal	DOI	Year	IF (2023)
1	Ankylosing Spondylitis Disease Activity Score (ASDAS): defining cutoff values for disease activity states and improvement scores	571	Machado P	Annals of the Rheumatic Diseases	10.1136/ard.2010.138594	2011	20.29
2	Anti-interleukin-17A monoclonal antibody secukinumab in treatment of ankylosing spondylitis: a randomized, double-blind, placebo-controlled trial	466	Baeten D	Lancet	10.1016/S0140-6736(13)61134-4	2013	98.4
3	2019 Update of the American College of Rheumatology/Spondylitis Association of America/Spondyloarthritis Research and Treatment Network Recommendations for the Treatment of Ankylosing Spondylitis and Nonradiographic Axial Spondyloarthritis	418	Ward MM	Arthritis & Rheumatology	10.1002/art.41042	2019	11.39
4	Decreased incidence of anterior uveitis in patients with ankylosing spondylitis treated with the antitumor necrosis factor agents infliximab and etanercept	302	Braun J	Arthritis and Rheumatism	10.1002/art.21197	2005	–
5	ASAS-EULAR recommendations for the management of axial spondyloarthritis: 2022 update	270	Ramiro S	Annals of the Rheumatic Diseases	10.1136/ard-2022-223296	2023	20.29
6	Risankizumab, an IL-23 inhibitor, for ankylosing spondylitis: results of a randomized, double-blind, placebo-controlled, proof-of-concept, dose-finding phase 2 study	252	Baeten D	Annals of the Rheumatic Diseases	10.1136/annrheumdis-2018-213328	2018	20.29
7	Ixekizumab, an interleukin-17A antagonist in the treatment of ankylosing spondylitis or radiographic axial spondyloarthritis in patients previously untreated with biological disease-modifying antirheumatic drugs (COAST-V): 16 week results of a phase 3 randomized, double-blind, active-controlled and placebo-controlled trial	247	van der Heijde D	Lancet	10.1016/S0140-6736(18)31946-9	2018	98.4
8	Switching TNF antagonists in patients with chronic arthritis: an observational study of 488 patients over a 4-year period	242	Gomez-Reino JJ	Arthritis Research & Therapy	10.1186/ar1881	2006	4.39
9	Efficacy and safety of upadacitinib in patients with active ankylosing spondylitis (SELECT-AXIS 1): a multicentre, randomized, double-blind, placebo-controlled, phase 2/3 trial	234	van der Heijde D	Lancet	10.1016/S0140-6736(19)32534-6	2019	98.4
10	The comparative 1-year performance of antitumor necrosis factor-alpha drugs in patients with rheumatoid arthritis, psoriatic arthritis, and ankylosing spondylitis: results from a longitudinal, observational, multicenter study	218	Heiberg MS	Arthritis and Rheumatism	10.1002/art.23333	2008	–

“Citations” indicates the total number of times the document has been cited globally. “Journal” refers to the journal in which the document was published. “DOI” represents the Digital Object Identifier, a unique identifier for the document. “Year” denotes the year of publication.

ASAS-EULAR = Assessment of SpondyloArthritis international Society-EULAR = European Alliance of Associations for Rheumatology, ASDAS = Ankylosing Spondylitis Disease Activity Score, COAST-V = clinical trial of anti-IL-17 in axial spondyloarthritis, DOI = Digital Object Identifier, IL-23 = interleukin-23, SELECT-AXIS 1 = study of upadacitinib in subjects with active ankylosing spondylitis 1.

**Figure 6.**
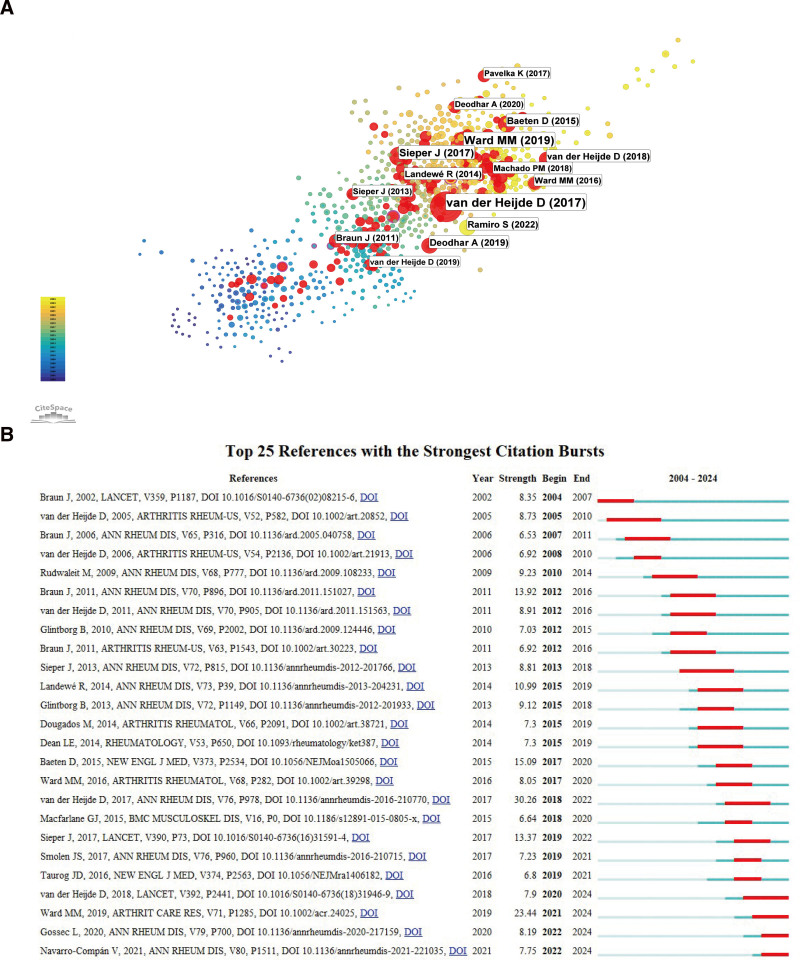
(A) Citation heatmap of referenced works. Nodes represent publications, with larger nodes indicating a higher frequency of citations. Nodes marked in red indicate references that exhibit a significant citation burst. (B) Top 25 references exhibiting the most significant citation bursts. The blue line represents the timeline, while the red segments show the periods when the references experienced bursts.

Figure 6 shows the citations of the cited references. In Figure 6A, nodes marked in red indicate references that exhibit a significant citation burst, identifying a total of 113 “hot references” (represented by 113 red nodes). Moreover, Figure 6B shows the 25 references with the most significant citation surges. The article with the highest strength and the most citations is a management recommendation for axSpA by van der Heijde et al, published in the Annals of the Rheumatic Diseases in 2017.^[20]^ This article has been cited 122 times and has a strength of 30.38. It emphasizes the use of biological disease-modifying antirheumatic drugs, including TNFis and IL-17 inhibitors, for patients who exhibit significant disease activity despite treatment with at least 2 NSAIDs. The second most cited reference is an updated recommendation article by Ward et al, published in 2019 in Arthritis & Rheumatology, which is also the third most frequently cited article overall. This reference has been cited 86 times and has a strength of 23.44. Additionally, Sieper et al, published in The Lancet in 2017, rank third and evaluate the effectiveness of NSAIDs and TNFis as treatments for axSpA, including AS.^[21]^ This paper also highlights the potential of IL-17 blockade as a novel and relevant treatment option. This reference has been cited 52 times, with an impact factor of 13.37. To summarize, the most frequently cited publications in AS biotherapy focus on clinical research, with key studies addressing disease activity assessment, the effectiveness of secukinumab, and updated treatment recommendations, highlighting the importance of TNF and IL-17 inhibitors.

#### 3.5.2. Keyword frequency and clustering analysis

Table 5 lists the 20 most frequently occurring keywords in biotherapy for AS. As shown in Table 5, rheumatoid arthritis and psoriatic arthritis are arthritis diseases often studied together with AS, with frequencies of 327 and 180 studies, respectively. This field focuses on the efficacy and safety of biologics in treating AS, their impact on patients’ quality of life, and the prevalence of other diseases. As illustrated in Table 5, infliximab, etanercept, and adalimumab are the most commonly studied biologics in treating AS, all targeting TNF-α.^[22]^

**Table 5:** The 20 most frequently occurring keywords in biotherapy for ankylosing spondylitis.

Rank	Keywords	Counts	Year	Centrality	Rank	Keywords	Counts	Year	Centrality
1	ankylosing spondylitis	654	2004	0.03	11	tumor necrosis factor	105	2005	0.09
2	rheumatoid arthritis	327	2005	0.04	12	etanercept	102	2006	0.08
3	psoriatic arthritis	180	2006	0.1	13	biological therapy	100	2006	0.05
4	efficacy	155	2005	0.07	14	arthritis	92	2007	0.09
5	double-blind	151	2005	0.1	15	disease	81	2005	0.07
6	axial spondyloarthritis	134	2012	0.04	16	quality of life	76	2005	0.1
7	therapy	129	2005	0.07	17	prevalence	69	2014	0.03
8	disease activity	122	2005	0.09	18	criteria	68	2011	0.03
9	infliximab	122	2004	0.05	19	adalimumab	66	2006	0.05
10	safety	119	2005	0.03	20	biologics	65	2008	0.06

“Counts” indicates the number of times the keyword appears. “Year” represents when the keyword first appeared in the literature. “Centrality” refers to the centrality of the keyword within the network of related terms, indicating its significance in the field.

**Figure 7.**
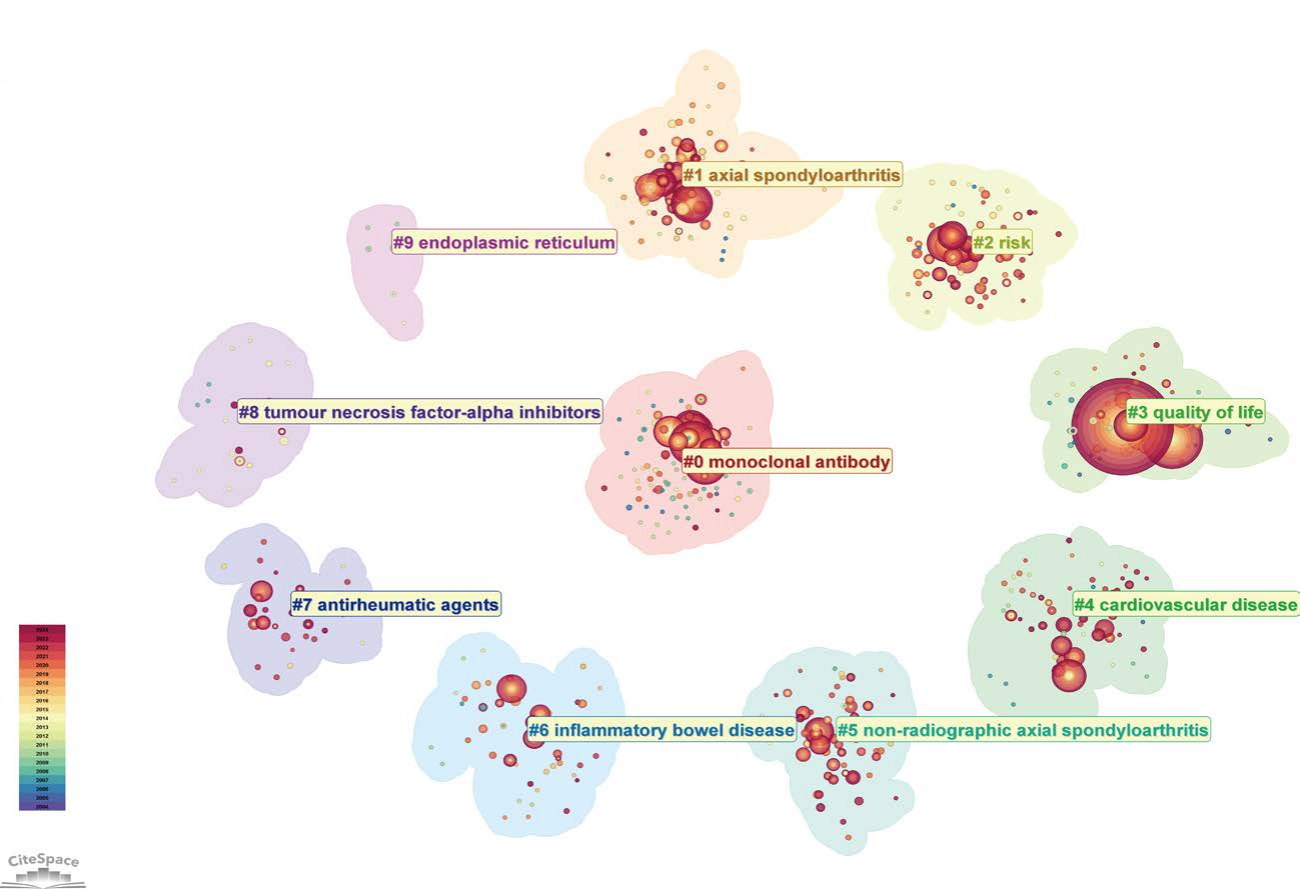
Keyword clustering in biologic therapies for AS (clustering method: Log-Likelihood Ratio). The size of the circles represents the frequency of keyword occurrences; larger nodes indicate higher frequencies. Different clusters contain distinct keywords and themes. Clusters with smaller label values encompass a broader range of keywords, indicating more diverse research content. Conversely, clusters with larger label values involve fewer keywords, suggesting a narrower research focus.

In this study, CiteSpace was used to cluster the keywords by selecting the “keyword” node type, resulting in a reasonable clustering graph with clustering module metrics of *Q* = 0.3334 and an average profile score of *S* = 0.7295 (Fig. 7). As shown in Figure 7, 10 major research clusters have been formed in the field of biotherapy for AS. The 10 clustering clusters are as follows: #0 monoclonal antibody, #1 axial spondyloarthritis, #2 risk, #3 quality of life, #4 cardiovascular disease, #5 nonradiographic axial spondyloarthritis, #6 inflammatory bowel disease, #7 antirheumatic agents, #8 tumor necrosis factor-alpha inhibitors, and #9 endoplasmic reticulum. Table 6 lists the details of the 10 clusters. The keyword content of each cluster indicates that this field mainly studies the impact of biological treatment for AS on patients’ quality of life, the effect of biologic therapy on patients” risk of other diseases, and the drug survival rate and predictors for AS patients after biologic treatment.

**Table 6:** Keyword content for 10 keyword clusters.

Cluster-ID	Number of sizes	Silhouette	Average year	Keywords content
0	99	0.694	2011	monoclonal antibody, double-blind, quality of life, placebo-controlled trial, golimumab
1	76	0.697	2013	axial spondyloarthritis, radiographic progression, anterior uveitis, drug survival, predictors
2	71	0.682	2015	risk, latent tuberculosis, persistence, rheumatology, inflammatory bowel diseases
3	66	0.806	2010	quality of life, disease activity, ankylosing spondylitis, rheumatoid arthritis, BASDAI
4	63	0.701	2015	cardiovascular disease, disease, network pharmacology, association, activation
5	58	0.662	2017	nonradiographic axial spondyloarthritis, fibromyalgia, criteria, clinical features, autoimmune disease
6	46	0.767	2015	inflammatory bowel disease, psoriatic arthritis, innovator infliximab, parallel group, rheumatic diseases
7	31	0.809	2019	antirheumatic agents, ankylosing, spondylitis, autoimmune diseases, health care
8	21	0.889	2013	tumor necrosis factor-alpha inhibitors, turnover markers, reproduction, antitumor necrosis factor-alpha, women
9	6	0.993	2010	endoplasmic reticulum, heavy chains, c terminal anchor, antigenic determinants, cell surface expression

“Cluster-ID” indicates the identification number assigned to each cluster of specific content. “Number of sizes” represents the number of keywords within each cluster. “Silhouette” denotes the silhouette score, which measures how similar an object is to its cluster compared to other clusters (a higher score indicates better clustering). “Average year” indicates the period of the research focus. “Keywords content” refers to the most representative keywords defining each cluster’s thematic content.

BASDAI = Bath Ankylosing Spondylitis Disease Activity Index.

#### 3.5.3. Keywords timezone analysis

**Figure 8.**
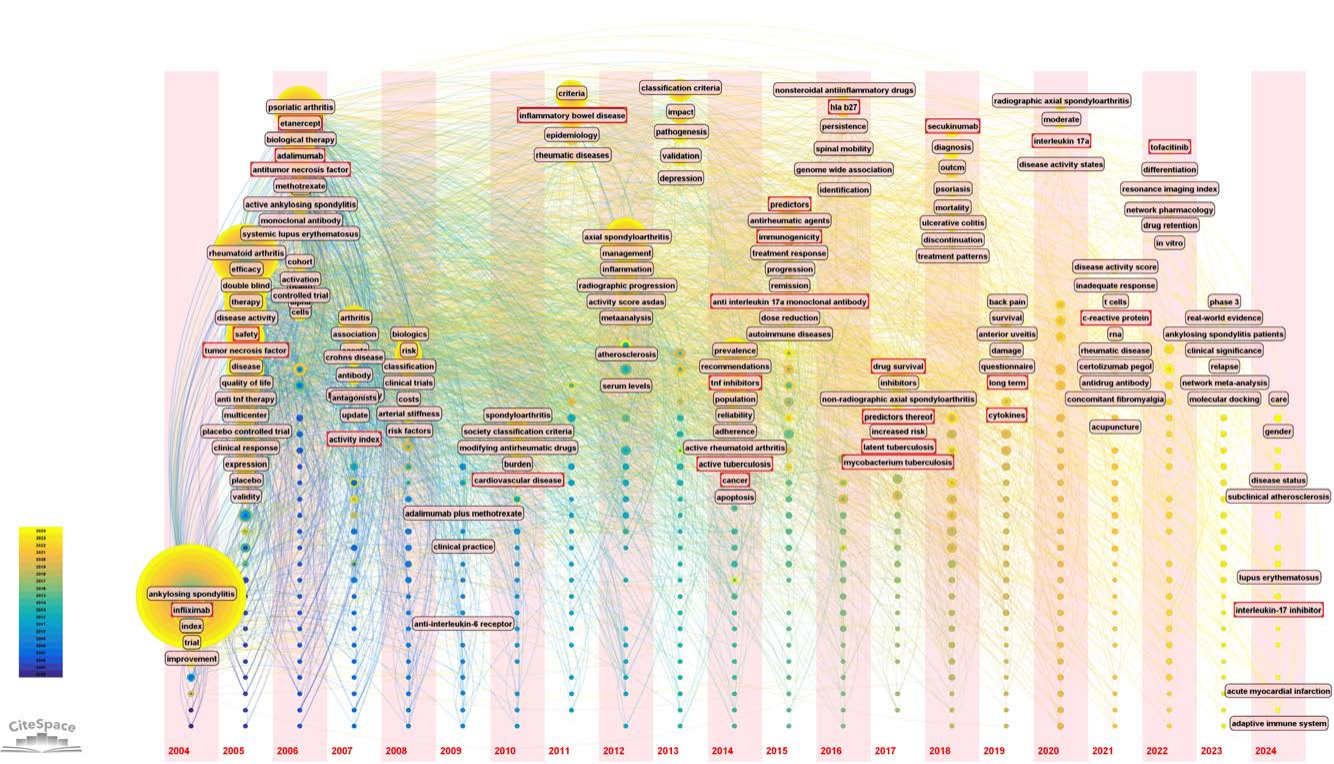
Visualization of the keyword time zone. Keywords are represented as nodes, with larger nodes signifying a higher frequency of occurrence, suggesting a greater volume of associated research on those keywords. The progression over time is depicted through a gradient of node colors. This gradient transitions from dark blue for earlier years to light blue, then green, and culminates in yellow for the most recent years (see the legend at the bottom left of the diagram). The keywords connected by the curves indicate co-researched, with thicker curves representing a higher frequency of joint studies. The connections between keywords in different columns illustrate the development of the research.

The time zone map can display the time dimension and the keywords, which provides us with a good understanding of the current trends in research within this area (Fig. 8). As illustrated in Figure 8, keywords that frequently appeared from 2004 to 2013 included “infliximab,” “efficacy,” “double-blind,” “safety,” “quality of life,” “adalimumab,” “etanercept,” and “anti-TNF therapy.” These keywords focused on the efficacy and safety of biologics targeting TNF-α in treating AS and axSpA, with randomized, double-blind experiments used as the primary research method. Starting in 2014, keywords shifted to “prevalence,” “predictors,” “drug survival,” “secukinumab,” and “HLA-B27,” illustrating that researchers in the field have begun to study the prevalence of other diseases associated with the use of biologics to treat AS, including tuberculosis and cancer. In addition, drug survival and biological predictors for treating AS are currently research hotspots. At this stage, attention has shifted from solely studying TNF inhibitors to exploring the effects of targeting interleukin and leukocyte differentiation antigens in treating AS. Between 2022 and 2024, terms such as “tofacitinib,” “drug retention,” and “gut microbiome” emerged successively. These indicate that the efficacy of Janus kinase (JAK) inhibitors in treating AS, the drug retention effects of biologics in AS treatment, and their impact on the gut microbiome of AS patients represent key research frontiers.

Table 7 presents the top 20 keywords with the most significant citation bursts. In this table, “Year” indicates when the keyword first appears, “Begin” denotes when the keyword starts to burst, and “End” indicates when the burst ends. The duration of the keyword bursts is visualized by the red line in the columns labeled “2004–2024.” As shown in Table 7, among the biotherapies for AS, infliximab had the most extended duration of the study (2004–2024), the most extended duration of the burst (2004–2015), and the greatest burst strength (12.54). The field was at its peak between 2005 and 2013. The burst keywords were “infliximab,” “work disability,” “etanercept,” “monoclonal antibody,” “antitumor necrosis fssssactor,” “quality of life,” “clinical practice,” “anti-TNF therapy,” and “rheumatoid arthritis.” The burst strengths for these keywords were 12.54, 3.48, 6.42, 5.07, 4.71, 3.6, 5.75, 4.19, and 3.07, respectively. At this stage, the primary research focuses on the influence of antitumor necrosis factor biologics (including infliximab and etanercept) on the quality of life and work disability of patients with AS. The keywords used for the 2014 to 2020 burst were “placebo-controlled trial,” “radiographic progression,” “methotrexate,” “immunogenicity,” “recommendations,” “predictors,” and “association.” Research has expanded to include the impact of immunogenicity on AS biotherapy, the effect of AS biotherapy on the radiographic progression of patients, the need for biotherapy following methotrexate treatment in patients with AS, and the association of AS with other diseases after biotherapy. Over the past 3 years, “pathogenesis,” “diagnosis,” “HLA-B27,” and “antirheumatic agents” have emerged as popular research topics. In conclusion, the time zone map and citation burst analysis reveal evolving research trends in AS treatment, shifting from TNF-α biologics efficacy and safety to drug survival, biological predictors, and the impact of biologics on gut microbiota and other diseases, with infliximab dominating early research and recent focus on JAK inhibitors and emerging topics like pathogenesis and HLA-B27.

**Table 7:**
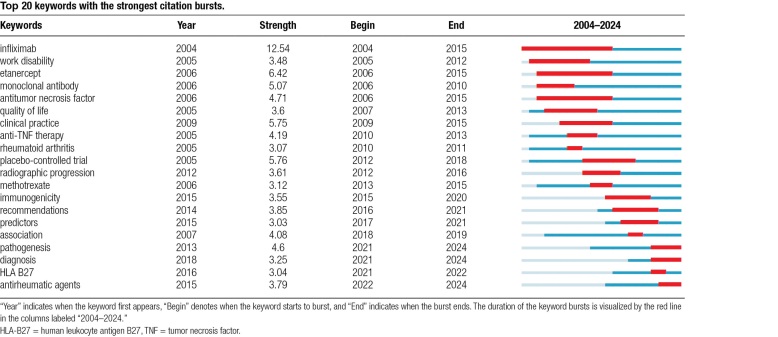
Top **20 keywords with the strongest citation bursts.**

## 4. Discussion

### 4.1. Basic information

This study employed bibliometric methods to perform a thorough and systematic analysis of research on biotherapy for AS. This analysis included annual publication counts, countries and regions involved, journals, authors, cited articles, cited references, and identifying research hotspots and frontiers. The study included a total of 975 publications on biotherapy for AS published in the Web of Science Core Collection and PubMed from January 1, 2004, to August 31, 2024. The steady increase in annual publications since 2012 signifies more than mere quantitative growth; it symbolizes the escalating scientific interest and the cumulative knowledge base that propels therapeutic advancements. This increasing trend tests the expanding understanding of AS pathophysiology and the relentless pursuit of more effective biological agents. The leading roles of the United States, China, and Germany in publication output underscore their pivotal positions in shaping the global research agenda for AS. Geographical distribution differences arise from several factors, primarily funding availability. Nations like the United States and Germany benefit from substantial government and private funding, enabling extensive clinical trials and biologics development.^[23]^ Regions with limited funding struggle to conduct impactful research, resulting in fewer publications. Additionally, disparities in research infrastructure affect outcomes, with advanced facilities and experienced researchers in developed countries more likely to achieve high-quality results.^[24]^ Clinical needs and disease prevalence, like the high rates of AS in specific populations, shape research priorities. A systematic review and network meta-analysis highlighted the dominant position of the United States in this field, making significant contributions to clinical trials and efficacy evaluations.^[25]^ The overlay visualization map of countries shows that nations, including Mexico, Brazil, Iran, Turkey, and France, have pioneered the study of AS biotherapy. More recently, countries such as Turkey, Japan, Bulgaria, and Argentina have substantially contributed to this subject (Fig. 3B). The journal that published the most significant number of articles in this field was Clinical Rheumatology, with 61 articles. It was followed by Rheumatology, which published 53 articles, and Rheumatology International, which published 48 articles. Moreover, the Annals of Rheumatism (5276 citations), the Journal of Rheumatism (2002 citations), and Arthritis and Rheumatism (1675 citations) ranked as the 3 periodicals with the highest total number of citations. These suggest the prominence of Clinical Rheumatology and other leading journals in publishing AS biotherapy research. Future studies should emphasize international collaboration and resource sharing to enhance global understanding and treatment of AS.

The 3 authors with the most significant number of publications in this field are Deodhar A, who has published 39 articles; Baraliakos X, with 33 publications; and van der Heijde D, with 32 publications. Their ongoing contribution to the field has shaped the research direction and influenced how the scientific community understands and treats AS. Our analysis found that specific keywords and publications have seen a surge in citations over the past 2 decades, indicating increased research interest and impact in biologic therapy for AS. This citation surge is primarily due to groundbreaking discoveries, such as identifying IL-17 as a crucial cytokine in AS pathogenesis, which led to the development of IL-17 inhibitors like secukinumab. These advances have introduced new treatment options and driven extensive research into their effectiveness and safety, resulting in numerous citations.^[26,27]^ The development of JAK inhibitors marks a significant advancement in treating AS, particularly for patients unresponsive to traditional biologics, boosting citations for related studies. Clinical trials have significantly contributed to this surge in citations.^[28]^ The randomized controlled trials on secukinumab and ixekizumab for AS have garnered significant interest and citations due to their potential to influence clinical practice.^[29–32]^ These trials strongly support the use of these biologics, impacting clinical guidelines and treatment protocols. Updates from authoritative bodies like the American College of Rheumatology and European Alliance of Associations for Rheumatology often incorporate new evidence, resulting in a surge of citations to foundational studies.^[19,33–37]^ These guidelines shape clinical practice and research, amplifying the impact of key publications. The surge in AS citations stems from groundbreaking discoveries, pivotal trials, and updated guidelines, underscoring the field’s dynamic nature and the need for ongoing innovation and evidence-based practice to enhance patient outcomes.

To sum up, research output in this field has increased significantly since 2012, highlighting the growing global interest in this area and the important contributions made by countries such as the United States, China, and Germany. Funding support and research infrastructure play a key role in publication trends. In integrating these data into a broader scientific context, we recognize that the distribution of research output and collaboration among researchers is not merely a reflection of academic productivity but also a critical factor in the velocity of scientific progress. At the same time, international cooperation and resource sharing must be strengthened to address disparities in research capabilities and further advance the understanding and treatment of AS.

### 4.2. Research hotspots

#### 4.2.1. Efficacy and mechanisms of biologics in AS: anti-TNF-α agents, anti-IL-17 agents, and JAK inhibitors

**Figure 9.**
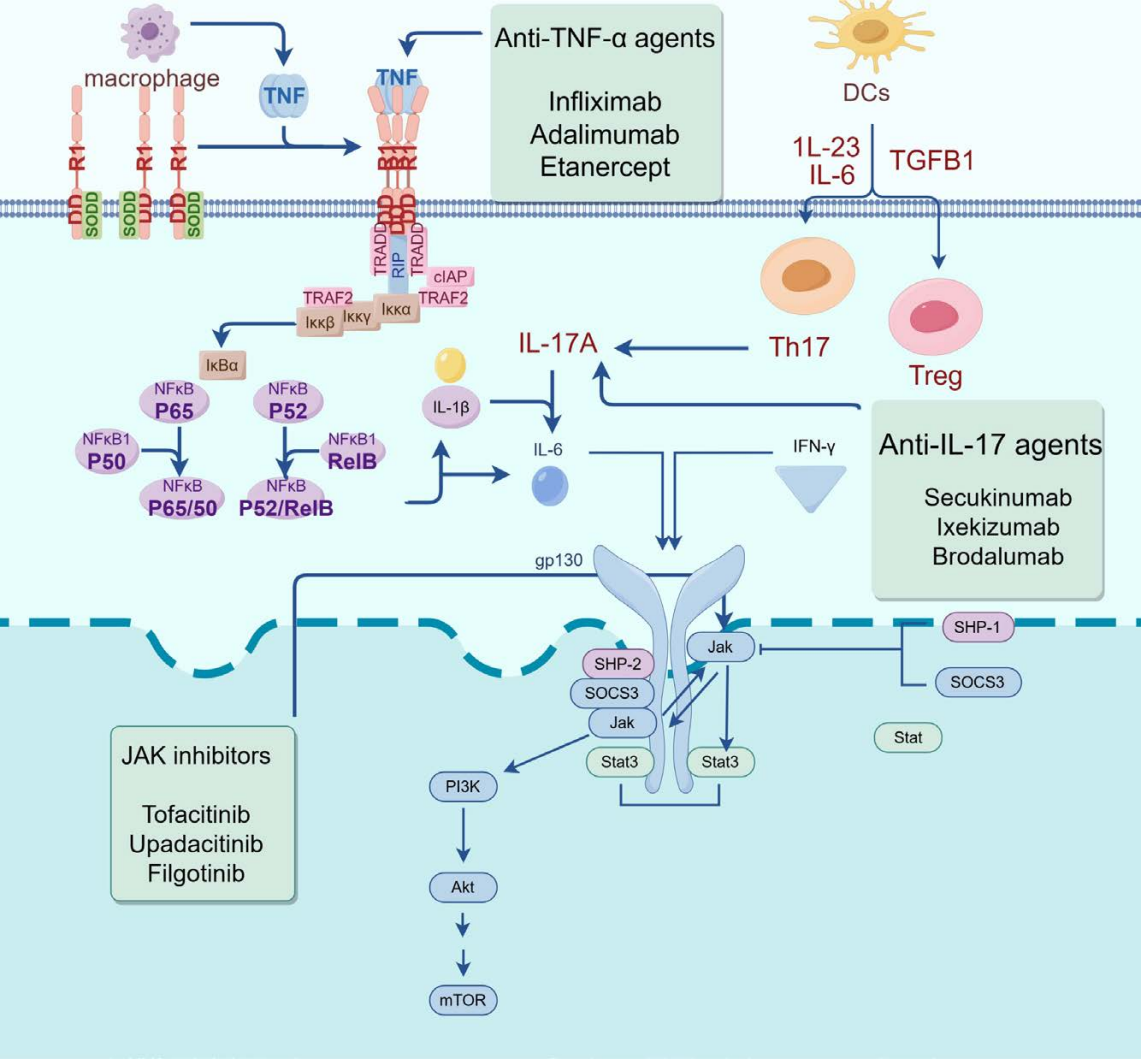
Schematic mechanism of antitumor necrosis factor-alpha drugs, anti-IL-17 drugs, and JAK inhibitors in treating ankylosing spondylitis. This diagram outlines how biological therapies for ankylosing spondylitis work. Anti-TNF-α drugs (like infliximab, adalimumab) reduce inflammation by blocking TNF signaling. Anti-IL-17 drugs (like secukinumab) inhibit IL-17A, impacting Th17 cells. JAK inhibitors (like tofacitinib and upadacitinib) disrupt JAK-STAT signaling, essential for immune activation. These therapies target key pathways in AS to ease symptoms and slow disease progression. Created by Figdraw. AS = ankylosing spondylitis, IL-17 = interleukin-17, JAK = Janus kinase, JAK-STAT = Janus kinase-signal transducer and activator of transcription, TNF-α = tumor necrosis factor-α.

Biological agents such as anti-TNF-α drugs, anti-IL-17 drugs, and JAK inhibitors have recently been proven effective in treating AS. Anti-TNF-α drugs, such as adalimumab, infliximab, and etanercept, reduce inflammation by inhibiting TNF-α and improving symptoms and quality of life. Studies have confirmed their efficacy, showing significant improvements in disease activity and functional status, as evidenced by decreased Bath Ankylosing Spondylitis Disease Activity Index (BASDAI) scores and increased Bath Ankylosing Spondylitis Functional Index scores.^[38,39]^ Anti-TNF-α drugs work by neutralizing the proinflammatory cytokine TNF-α, blocking its receptor binding, and potentially reducing other inflammatory mediators.^[40]^ While effective in alleviating symptoms of AS, their impact on slowing spinal imaging progression is debated, suggesting a need for combination therapies.^[41]^ Despite generally good tolerance, these drugs require monitoring for possible immunogenic reactions and other side effects.^[42]^ When treating AS with anti-TNF-α drugs, monitoring patient responses closely and tailoring treatments to individual needs is crucial. In recent years, biologic therapies targeting IL-17, a proinflammatory cytokine integral to the pathophysiological mechanisms of AS, have demonstrated substantial efficacy in managing this condition. Research indicates that IL-17 is produced by T cells, and its secretion is contingent upon several upstream cytokines, including IL-23.^[43]^ In the therapeutic context of AS, IL-17 inhibitors such as secukinumab and ixekizumab have proven effective. These agents mitigate joint inflammation and enhance patients’ quality of life by inhibiting IL-17A activity and are generally well tolerated.^[44]^ Additionally, newer agents, such as brodalumab, which targets the IL-17 receptor, have exhibited promising efficacy.^[45]^ The effectiveness of IL-17 inhibitors has been corroborated through numerous clinical trials. For example, a systematic review and meta-analysis revealed that IL-17A inhibitors significantly improved response rates in the Assessment of SpondyloArthritis international Society (ASAS) criteria (ASAS20 and ASAS40 response) among AS patients.^[46]^ Dual neutralization of IL-17A and IL-17F can prevent inflammation-driven bone formation, suggesting that IL-17 plays a crucial role in AS. Despite the impressive efficacy of IL-17 inhibitors, further research involving larger sample sizes and extended follow-up periods is necessary to assess their safety profile thoroughly. JAK inhibitors are also promising as novel AS treatments because they reduce inflammation via the JAK-signal transducer and activator of transcription signaling pathway. Compared with placebo, JAK inhibitors such as tofacitinib, upadacitinib, and fingolitinib have proven effective in treating AS, significantly increasing ASAS20 and ASAS40 response rates and disease activity, quality of life, and magnetic resonance imaging outcomes. Safety concerns remain, particularly regarding infection risk; more studies are needed to confirm their long-term safety and efficacy in the clinic.^[47–49]^ To sum up, biological agent therapy (including anti-TNF-α drugs, anti-IL-17 drugs, and JAK inhibitors) has shown significant efficacy in treating AS by reducing inflammation and improving the quality of life. However, although these treatment regimens are generally well tolerated, their long-term safety remains a continuous concern, especially regarding the risk of infection and the impact on the progression of spinal imaging. Further research is crucial for addressing these issues and optimizing the treatment strategies for patients with AS. Diagram illustrating the treatment mechanism for AS using biological anti-TNF-α drugs, anti-IL-17 drugs, and JAK inhibitors (Fig. 9).

#### 4.2.2. Personalized treatment of biologics in AS

Owing to varied patient responses, personalized therapy is crucial. Biomarkers such as C-reactive protein, patient global assessment, and the BASDAI can predict the efficacy of TNF inhibitors. The Bath Ankylosing Spondylitis Functional Index and body mass index are also linked to treatment response.^[50]^ The body’s ability to produce antidrug antibodies, or immunogenicity, impacts the effectiveness and safety of biological treatments. Testing for immunogenicity before treatment can help optimize therapy, as it may reduce efficacy and increase side effects.^[51]^ A study revealed that male patients, those with HLA-B27 positivity, and those starting biologics later tend to have better drug survival, suggesting that these factors could predict biologic efficacy and guide personalized treatment.^[52]^ Additionally, network meta-analyses indicate that biologics vary in clinical efficacy and safety, with infliximab showing greater efficacy at 12 and 24 weeks.^[53]^ Selecting the right biologic for AS requires considering the patient’s unique situation and the drug’s properties. In summary, personalized therapy is crucial, and using biomarkers can enhance the prediction of patient response, increasing treatment effectiveness and cost efficiency. Although existing biomarkers like C-reactive protein, BASDAI, and immunogenicity testing can predict treatment responses, more research is needed to identify new biomarkers and refine personalized treatment strategies.

#### 4.2.3. Combined use of biologics and other therapeutic approaches

Biological agents can be combined with traditional treatments such as NSAIDs and physiotherapy to increase therapeutic efficacy, reduce side effects, and enhance patient quality of life. For example, integrating personalized physical therapy and rehabilitation equipment with biological treatments can aid in joint function recovery. Recent advances in AS treatment, particularly biologics, highlight the potential of combined therapies. Research shows that biologics, such as TNF and IL-17A inhibitors, significantly improve the quality of life in AS patients.^[54]^ Combining biologics with NSAIDs, immunomodulators, or nonpharmacologic therapies such as hydrotherapy can improve the treatment of AS.^[55]^ Anti-TNF monoclonal antibodies and JAK inhibitors have shown significant efficacy in improving diseases when combined with other drugs. Optimizing therapeutic strategies through therapeutic drug monitoring can enhance anti-TNF therapy and reduce complications.^[56]^ Patient quality of life is a crucial factor in evaluating combination therapies. Research indicates that the administration of biologics, such as ixekizumab, markedly enhances clinical symptoms and quality of life in patients with AS, particularly those who exhibit inadequate response or intolerance to TNF inhibitors.^[57]^ Furthermore, integrating biologics with other therapeutic modalities has demonstrated the potential to increase patients’ quality of life and work productivity.^[58]^ In conclusion, by optimizing treatment regimens and integrating multiple therapies, therapeutic efficacy can be further improved, adverse effects can be minimized, and patient quality of life can be substantially enhanced. Of course, although studies have shown that these combined treatments can lead to better clinical outcomes, further research is still needed to determine the optimized strategies for integrating multiple treatment modalities and to address the potential cognitive gaps regarding the long-term effects and safety of such combined treatments.

#### 4.2.4. Analysis of long-term safety and adverse effects of biologics in patients with AS

The safety of biologics for treating AS is crucial due to the long-term nature of treatment. While TNF-α inhibitors, IL-17 inhibitors, and JAK inhibitors are effective, they can cause adverse effects over time. TNF-α inhibitors like infliximab and etanercept, known for their immunomodulatory effects, may raise the risk of infections, including tuberculosis and opportunistic infections, because of their broad immunosuppressive nature.^[59,60]^ Prolonged drug use can lead to autoimmune diseases and cancer, though these cases are relatively rare.^[61,62]^ Furthermore, drug-resistant antibodies decrease efficacy and cause infusion reactions.^[63,64]^ IL-17 inhibitors like secukinumab effectively treat AS but can increase the risk of infections, especially in the upper respiratory tract, and may worsen inflammatory bowel disease in some people.^[65,66]^ Concerns persist about chronic immunosuppression and its effects on immune function despite ongoing long-term data on IL-17 inhibitors.^[67]^ JAK inhibitors like tofacitinib are promising for AS treatment. However, they have significant side effects, including infection risk, thromboembolic events, potential liver damage, and, with long-term use, possible cardiovascular issues and cancer.^[68–72]^ In summary, while biologics benefit AS, their long-term use requires careful monitoring for adverse effects. Future research should clarify their long-term safety and identify biomarkers to reduce risks and enhance treatment.

### 4.3. Research trends

#### 4.3.1. Development of novel biologics that target dual pathways in AS

In recent years, extensive research into the pathophysiological mechanisms of AS has facilitated notable advancements in developing novel biological therapies. Dual-targeted biologics, an innovative therapeutic approach, are designed to concurrently target 2 distinct proteins implicated in the pathogenesis of AS, thereby enhancing therapeutic efficacy while reducing adverse effects. Empirical studies have demonstrated dual-targeted biologics’ potential in preclinical and clinical assessments.^[73]^ Network meta-analyses have indicated that biologics exhibit substantial clinical effectiveness in treating AS; however, there is a paucity of direct comparative studies among different biologics. Through indirect comparisons, it has been determined that infliximab has the lowest number needed to treat to achieve an additional ASAS20 and ASAS40 responses, indicating high clinical efficacy.^[74]^ Despite the favorable efficacy of biologics in the management of AS, approximately 20% to 30% of patients exhibit suboptimal responses to TNF-α inhibitors, necessitating the development of innovative biologic therapies.^[75]^ On a global scale, research on the application of biologics in AS treatment has shown a consistent upward trend, focusing on JAK inhibitors, advancements in spinal imaging, biosimilars, and assessing adverse events emerging as prominent areas of investigation.^[76]^ These studies pave the way for novel directions and opportunities in the future management of AS. The advent of dual-targeted biologics has introduced a new paradigm of hope in AS treatment. By concurrently targeting multiple inflammatory pathways, these advanced agents are anticipated to enhance therapeutic efficacy while minimizing adverse effects, offering more personalized and effective treatment modalities for AS patients. Future research endeavors need to elucidate further the novel biologics’ role in AS management and their influence on imaging progression.

#### 4.3.2. Development of novel biologics targeting new cytokine pathways in AS

Biological agents, particularly those that target specific cytokines, have emerged as significant therapeutic options for AS. TNF-α inhibitors are pioneering biologics that have been demonstrated to be effective in treating AS and successfully managing inflammatory pain and peripheral clinical manifestations in the spine.^[77]^ More recently, biologics that target IL-17 and IL-23 have exhibited potential in the treatment of AS.^[78]^ Notably, secukinumab, an anti-IL-17A monoclonal antibody, has demonstrated efficacy in slowing the radiological progression of AS.^[79]^ Furthermore, JAK inhibitors, a novel class of oral synthetic drugs, have shown promise in AS management. These pharmacological agents mitigate the inflammatory response by inhibiting the JAK-signal transducer and activator of transcription signaling pathway, alleviating patient symptoms. In addition to these innovative biologics, research has indicated a potential causal link between the gut microbiota and AS, suggesting that specific bacterial taxa may influence the progression of AS by modulating inflammatory cytokines such as IL-23 and interferon gamma.^[80]^ This insight offers a novel therapeutic avenue, focusing on the modulation of the gut microbiota to alter the trajectory of AS. In summary, as the understanding of AS pathophysiology has advanced, the emergence of novel biologics has expanded the therapeutic arsenal available to AS patients. These agents aid in symptom management and may contribute to decelerating disease progression. Nonetheless, further clinical investigations are imperative to substantiate these treatments’ long-term efficacy and safety.

#### 4.3.3. Gene therapy techniques for AS

Gene therapy techniques have demonstrated potential in treating AS by correcting disease-associated genetic defects through modifying or replacing specific genes. As technological advancements continue, gene therapy matures and may provide novel solutions for treating diseases such as AS.^[81]^ In Japan, advancements in gene editing technology have led to significant breakthroughs in gene therapy despite potential risks such as unintentional modification or deletion of gene sequences.^[82]^ Within the pathophysiology of AS, polymorphisms in the endoplasmic reticulum aminopeptidase 1 gene are closely linked to disease susceptibility. These polymorphisms, which play crucial roles in the processing and presentation of antigenic peptides, may influence the pathogenesis of AS.^[83,84]^ Furthermore, transcription factors such as T-box expressed in T cells and runt-related transcription factor 3 are crucial in T-cell development and function, with polymorphisms in these genes potentially offering novel targets for AS treatment.^[85]^ Although the close association between HLA-B27 and AS has been studied, the genetic mechanism of AS is not yet fully understood.^[86]^ Furthermore, the commercialization of gene therapy technology was previously hindered, mainly due to the gap between technological maturity and capital investment. Although gene therapy applications for AS remain nascent, their potential significance should not be underestimated. Future research should focus on elucidating the genetic and epigenetic mechanisms underlying AS to increase the understanding and management of this disease.^[87]^ By integrating genomic and epigenomic methodologies, researchers can more comprehensively elucidate the pathogenesis of AS, thereby informing the development of innovative therapeutic strategies.^[88]^ To summarize, gene editing technologies enable the genetic modification of a patient’s immune cells, increasing their ability to recognize and target the aberrant cells or molecules responsible for AS, thereby achieving therapeutic objectives. Future research should clarify the genetic and epigenetic factors involved in AS to enhance therapeutic strategies. Nevertheless, the current applications of gene therapy are still in early development and require further investigation to realize their full potential.

#### 4.3.4. Stem cell therapy techniques for AS

Stem cell therapy is increasingly recognized as a promising therapeutic strategy. By leveraging stem cells’ self-renewal and pluripotent differentiation capabilities, these cells are transplanted into patients to facilitate the repair of damaged joint tissues and modulate immune system functions. Mesenchymal stem cells (MSCs), in particular, exhibit immunomodulatory and anti-inflammatory properties, which can mitigate the inflammatory response in AS through mechanisms such as the secretion of anti-inflammatory factors and the inhibition of excessive immune cell activation. Research indicates that MSCs are integral to immunomodulation and bone formation and that their pluripotent differentiation potential and immunomodulatory functions render them promising candidates for AS treatment. Nonetheless, the immunomodulatory and osteogenic capacities of MSCs in AS patients may be influenced by genetic predispositions, the internal environment, infections, and mechanical forces, potentially contributing to disease progression.^[89]^ Furthermore, oxidative stress-induced mitochondrial dysfunction has been implicated in promoting MSC senescence in AS, which may further aggravate inflammation and disease progression.^[90]^ Understanding the impact of oxidative stress on the functionality of MSCs in patients with AS is essential for developing novel therapeutic strategies. In stem cell therapy, the application of induced pluripotent stem cell (iPSC) technology has been explored in AS research. By deriving iPSCs from AS patients’ peripheral blood mononuclear cells, researchers can establish a platform to investigate the pathological mechanisms underlying new bone formation in HLA-B27-positive AS patients.^[91]^ This cellular model offers valuable insights into the pathophysiology of AS and lays the groundwork for future therapeutic interventions. Although current investigations have focused primarily on animal models and preliminary clinical trials, stem cell transplantation has demonstrated potential in reducing inflammation and enhancing the quality of life of patients.^[92]^ Stem cell therapy shows promise for treating AS, but further clinical research is needed to confirm its safety and effectiveness. Understanding the mechanisms of MSCs and iPSCs in AS could guide future treatment strategies.

#### 4.3.5. Impact of biologics on the gut microbiota in AS

Research indicates that the gut microbiota is integral to the pathogenesis of AS, and biological therapies may exert therapeutic effects by modulating these microbial communities. Evidence suggests that the gut microbiota composition of AS patients significantly diverges from that of healthy individuals, notably characterized by a decreased abundance of short-chain fatty acid-producing bacteria. Biological interventions, such as anti-TNF-α therapy, have partially restored the gut microbiota in AS patients, aligning it more closely with the microbial profile of healthy controls.^[93]^ In particular, there was a significant increase in the abundance of short-chain fatty acid-producing bacteria, such as *Megamonas* and *Lachnoclostridium*, in patients with AS following anti-TNF-α treatment, which was negatively correlated with disease severity.^[94]^ Moreover, research indicates that biological therapies may ameliorate AS symptoms by modulating gut microbiota diversity. Patients with AS receiving biologic therapies demonstrate increased gut microbiota diversity, which is strongly associated with treatment efficacy.^[95]^ For example, the alpha diversity index of the gut microbiota increased in patients receiving biologic therapies, implying that the microbiota composition more closely resembles that of a healthy population.^[96]^ Biological therapies influence not only bacterial communities but also intestinal viral populations. Research has demonstrated that the intestinal viral populations in patients with AS are significantly altered compared with those in healthy controls and that biological therapies may modulate these viral populations.^[97]^ Consequently, the therapeutic effects of biotherapies in AS are mediated through direct immunomodulatory actions and by altering the composition and function of the gut microbiota. This insight offers new perspectives and potential therapeutic targets for AS treatment. Future research should investigate the specific mechanisms of action and the clinical applicability of these microbiota further.

#### 4.3.6. Interdisciplinary research methods and their impact on AS

Integrating interdisciplinary research, including genetics, microbiome studies, and immunology, enhances our understanding and treatment of AS. Genetic research on HLA-B27 and endoplasmic reticulum aminopeptidase 1 polymorphisms has identified susceptibility genes that could lead to personalized therapies.^[98–100]^ Studies of the gut microbiome show that patients with AS have a less diverse microbial profile and different levels of certain bacteria compared to healthy individuals.^[101,102]^ Modulating gut microbiota with diet or probiotics could offer new treatments. Understanding cytokines like IL-17 and TNF-α in inflammation has also led to targeted biologic therapies.^[103,104]^ Integrating interdisciplinary methods enhances our understanding of AS and leads to more effective personalized treatments. Future research should focus on the interaction between genetics, microbiome, and immune response to develop innovative management strategies and improve patient outcomes.

### 4.4. Limitations

This bibliometric analysis of biologic therapies for AS highlights limitations in current research. Studies often focus on the short-term efficacy of TNF-α, IL-17, and JAK inhibitors, overlooking long-term effects crucial for chronic conditions. They also lack diverse patient populations and consideration of comorbidities, limiting treatment personalization. Additionally, research on biologics’ impact on gut microbiota and its role in disease progression is scarce. This analysis addresses these gaps by exploring the microbiota’s microbiota’s role in AS, evaluating existing literature, identifying trends, and suggesting future research directions. This study underscores the necessity for long-term research on the efficacy and safety of biologics, personalized treatments, and their impact on gut microbiota. It outlines future research directions, calling for broader patient studies and a deeper understanding of microbiota’s role in disease. The study relied on the Web of Science Core Database and PubMed, possibly limiting dataset completeness and excluding relevant studies from other sources. Bibliometric limitations may overlook emerging trends, and strict screening might omit important findings, potentially biasing publication types. Despite these issues, the analysis identifies key trends and stresses the need for further research to address knowledge gaps in AS treatment.

### 4.5. Future perspectives

Our study shows progress in biologic therapies for AS, but key research gaps remain. Addressing these is crucial for enhancing efficacy, patient outcomes, and the broad use of biologics. First, new therapeutic targets and combined treatment strategies. Current studies primarily focus on the TNF-α, IL-17, and JAK pathways, but the complex pathophysiology of AS indicates that other cytokines and pathways are equally important. Future research should prioritize discovering new therapeutic targets and exploring dual-target biological agents and new cytokine pathways. In addition, optimizing combined treatment strategies, such as pairing TNF inhibitors with other biologics or targeted synthetic DMARDs, can significantly improve patients’ quality of life.^[75,105]^ An in-depth study of the impact of the IL-23/T helper cell 17 pathway and biologics on the gut microbiota will provide better treatment options for patients who do not respond to existing therapies.^[27,106]^ Second, personalized treatment and biomarker development. The genetic markers of AS can identify high-risk groups and customize treatment plans, which is crucial for achieving precision medicine.^[107–109]^ Due to the differences in therapeutic effects among different patients, there is an urgent need to develop biomarkers to predict treatment responses and customize treatment plans. Interdisciplinary research, combined with technologies such as machine learning, will help enhance the understanding of biotherapy, achieve precise and personalized treatment, and thereby improve patient treatment outcomes and options.^[108]^ Third, the potential and challenges of emerging technologies. Emerging technologies such AS gene editing and stem cell therapy may revolutionize the treatment of AS by curing diseases and reducing the demand for drugs.^[110,111]^ However, these technologies still face technical and ethical issues. Future research should assess the feasibility and safety of these emerging technologies while addressing the related ethical and legal issues. Fourth, economic and accessibility issues. The high cost of biological agents is the main obstacle restricting their broad application, especially for low- and middle-income countries. Future research should assess the long-term cost-effectiveness of biological agents, develop more cost-effective biosimilars, and explore alternative funding sources to increase accessibility.^[74]^ Furthermore, studying the economic impact of biological agents on the healthcare system and patients’ quality of life is vital to policymakers and medical decision-makers. By filling these research gaps, we can develop more effective, targeted, and accessible methods for treating AS, which will benefit patients more.

## 5. Conclusion

This paper uses bibliometric methods to analyze research trends in biologics for AS from 2004 to 2024. The attention this field receives is rising daily. The United States, China, and Germany are leading in publications, among which the United States has particularly advanced the progress of clinical trials. Clinical rheumatology published more articles, whereas Annals of Rheumatic Diseases had the most citations. Research has focused on the effectiveness and mechanisms of biological agents, personalized treatments, and combination therapies. Anti-TNF-α, anti-IL-17, and JAK inhibitors show promise for AS treatment, but their impact on slowing spinal imaging progression is debated. Identifying biomarkers to predict biological efficacy is crucial for personalized therapy. Combining biologics with conventional treatments can enhance effectiveness and reduce side effects. Future research will focus on developing biologics targeting dual and neo-cytokine pathways and gene and stem cell therapies. The impact of biologics on the gut flora is also gaining scholarly interest, offering fresh insights into AS treatment. Despite data limitations, this study highlights current research trends in biologics for AS and guides future research directions.

## Acknowledgments

We sincerely appreciate the constructive feedback provided by the anonymous reviewers. Thanks to Figdraw (HOME for Researchers) for assisting with the mechanism map (ID: UASAP15556).

## Author contributions

**Funding acquisition:** Chang-wen Quan.

**Investigation:** Xin-xin Zhong.

**Software:** Fu Shen.

**Validation:** Fu Shen.

**Writing – original draft:** Rong Deng, Xuan Jiang.

**Writing – review & editing:** Feng Qian, Chang-wen Quan.
